# Effect of Wild and Cultivated Rice Genotypes on Rhizosphere Bacterial Community Composition

**DOI:** 10.1186/s12284-016-0111-8

**Published:** 2016-08-24

**Authors:** Matthew Shenton, Chie Iwamoto, Nori Kurata, Kazuho Ikeo

**Affiliations:** 1 Plant Genetics Laboratory, National Institute of Genetics, 1111 Yata, Mishima, Shizuoka 411-8540 Japan; 2 Laboratory for DNA Data Analysis, National Institute of Genetics, 1111 Yata, Mishima, Shizuoka 411-8540 Japan; 3 Department of Genetics, School of Life Science, Graduate University for Advanced Studies, 1111 Yata, Mishima, Shizuoka 411-8540 Japan

## Abstract

**Background:**

Deposition and secretion from roots influences the composition of the microbial communities surrounding them in the rhizosphere, and microbial activities influence the growth and health of the plant. Different host plant genotypes result in differences in those microbial communities. Crop genomes may have a narrow genetic base because of bottlenecks that occurred when domesticated crops were derived from small populations within the progenitor species. Desirable traits influencing root-associated microbial communities might therefore have been lost in the transition from wild species to modern cultivars. To investigate the diversity of bacterial communities associated with wild and cultivated rice, we surveyed a series of plant species and cultivars spanning the *Oryza* genus, growing them in the same nutrient-poor soil and assessing the bacterial composition of their rhizospheres and the surrounding soil using 16S rDNA sequencing.

**Results:**

Root-associated bacterial communities showed small but significant differences dependent on the plant genotype. We found that differences between bacteria associated with differing plant genotypes were only weakly correlated with the phylogenetic distance between the *Oryza* wild species and cultivars. In ordination plots, domesticated and wild samples could be separated on the basis of their associated bacterial communities. Taxa of the Anaerolineae were overrepresented in wild samples compared to domesticated ones. Certain methanotrophs were overrepresented in the earliest diverged part of the *Oryza* genus.

**Conclusions:**

Bacterial populations associated with the rhizosphere of wild rice species displayed differences with those associated with cultivars, suggesting that root traits selected in domestication could have significant influence on the rhizosphere microbiota composition. Variation within the genus seems to influence the representation of methanotrophs. This suggests that greenhouse emissions from paddy fields could be altered by manipulating plant genotypes through the introgression of wild rice genetic material.

**Electronic supplementary material:**

The online version of this article (doi:10.1186/s12284-016-0111-8) contains supplementary material, which is available to authorized users.

## Background

The term rhizosphere was first defined by Hiltner in 1904 (Hiltner 1904; reviewed in Hartmann et al. 2008), and can be understood as a region of increased microbial growth surrounding the roots that is dependent on plant exudates. Even more than 100 years ago, Hiltner recognized the profound importance of the rhizosphere composition for plant health, growth and yield.

There has been a recent increase in interest in the properties of soil-associated microbiota driven by the relative ease of quantifying microbes based on metagenomics and next generation sequencing (Lakshmanan et al. 2014). It has also been theorized that one direction in crop improvement would be to improve low input cultivation via optimization of microbial activities in the soil (Quiza et al. 2015). The assembly of crop-associated microbial communities results from complex plant genome x metagenome x environment interactions resulting in functional microbial physiological activities in the root, at the root surface and in the rhizosphere. Because microbial populations associated with plant roots depend on the genotype of the host plant (reviewed in Berg and Smalla, 2009), the functions of the plant associated microbiota might be optimized by changing the plant genotype via plant breeding.

The compositions of various plant rhizospheres have been investigated in recent years. Bulgarelli et al. (2012) and Lundberg et al. (2012) showed that rhizosphere communities are dominated by members of the Actinobacteria, Bacteroidetes, and Proteobacteria and are strongly influenced by soil type with quantitative variations among plant genotypes, and that the rhizosphere consists of bacteria derived from the bulk soil as well as saprophytic bacteria that metabolize plant or root debris. In rice, Knief et al. (2011) defined functions in the rhizosphere and phyllosphere, and Edwards et al. (2015) showed variations in the rhizosphere among genotypes of the indica and japonica subspecies of *Oryza sativa* and of the cultivated African rice *O. glaberrima*. Similarly, Peiffer et al. (2013) found a small significant effect of maize genotype in modern inbred maize cultivars on the alpha and beta diversity of microbial communities in the rhizosphere.

The effects of domestication and breeding on plant physiology and development have likely had a strong influence on the microhabitats inhabited by root-associated microbes. Plant architecture has been radically altered during domestication. While changes in the aerial parts are more obvious, root phenotypes have also likely changed, although their selection may have been indirect — for example via selection for drought avoidance, flooding tolerance or yield traits. Such changes would likely affect microbial populations associated with rice roots.

Few studies have considered the impact of domestication or crop improvement on root-associated microbes. In barley, Bulgarelli et al. (2015) studied root-associated microbes of a modern variety, a landrace, and a wild accession, finding a small but significant effect of the plant genotype on the root microbial communities. Szoboszlay et al. (2015) assessed rhizosphere processes of ancestral and domesticated corn varieties, also finding a small influence of plant genotype on the rhizosphere.

In addition to root architecture or microhabitat, rhizosphere and especially rhizoplane and endosphere composition may be influenced by root exudates and the plant immune system. For example, Carvalhais et al. (2015) investigated the importance of jasmonic acid signaling and associated changes in exudation for the Arabidopsis rhizosphere microbial communities. Given that some immune system components appear highly variable within the *Oryza* genus (Jacquemin et al. 2013), it would be interesting to observe genus-wide variability of root-associated microbial communities.

*Oryza* species genomes are categorized on the basis of the ability of their chromosomes to pair correctly during meiosis in interspecies F1 hybrids (Kurata 2008). All cultivated species and their wild progenitors are diploid and are among the species possessing the A genome (AA). Other wild species have different genome types. *O. brachyantha* is the earliest diverged diploid species having an FF genome type (Vaughan et al. 2008). The *Oryza* genus is also divided into various species complexes. The two largest are the Oryza sativa complex, which comprises all cultivated species, their wild relatives and other species possessing the AA genome type; and the Oryza officinalis complex, which also has pan-tropical distribution and includes BB, CC, DD, and EE genomes in diploid and allotetraploid combinations (Vaughan et al. 2008). The early diverging genus members used in this study: *O. brachyantha*, *O. longiglumis* and *O. granulata* are referred to herein as “early-diverged” members of the genus.

We surveyed a wide range of wild and cultivated *Oryza* species and accessions from across the genus using 16 s rDNA sequencing as an indicator of the representation of root-associated and soil living bacteria. We aimed to discover how closely the variation in rhizosphere bacterial communities reflected the phylogeny of the *Oryza* genus, whether there were exceptional wild *Oryza* species with unique patterns of associated bacteria and whether domestication had a significant effect on the bacterial communities hosted by rice roots.

## Methods

### Plant and Soil Materials

Rice and wild rice strains were obtained from the wild rice collection at the National Institute of Genetics, Japan. The hulls were removed from the seeds and 15 seeds from each line were sterilized by immersion in 70 % ethanol 0.1 % Triton X-100 for 1 min, followed by 10 min in 2 % sodium hypochlorite solution. Seeds were washed three times for 1 min in sterile water and incubated for 48 h on ½ MS plates containing 2 % sucrose before transferring to pots.

Soil was collected from an experimental paddy field that had been planted for 3 years with *Oryza sativa* but had received no fertilizers or pesticides. Soil was collected on April 10^th^, 2014 from the upper 15 cm of the soil from 3 separate locations within the field. The soil was mixed and passed through a 2 mm mesh sieve to remove stones, debris and plant material. Soil was placed into flexible 18 cm diameter polythene pots to a depth of 15 cm. The pots were placed into large trays and water was maintained in the trays at a level 2 cm below the level of the soil. Five germinated seeds were placed in each pot. Each *Oryza* accession was represented by three pots which each contained five seeds, and the pots were arranged in a randomized block design.

Plants were grown in the greenhouse for 4 weeks. Due to poor germination or survival of some wild rice species, the number of live plants remaining in each pot at the sampling time varied between one and five.

The pots were cut and the sides of the pot were removed. Each of the rice seedlings was lifted onto a clean piece of saran wrap and shaken to remove soil loosely adhering to the roots. This soil was stored as the loosely attached soil sample. The roots were then transferred to a sterile sampling bag and squeezed with a soft-headed hammer in order to release root-associated/endophytic bacterial cells. The rice roots were then removed leaving a mixture of adhered rhizosphere soil, rhizoplane material and endophytic material. This sample was taken as rhizosphere. Unplanted pots containing soil treated in the same manner as the planted pots were cut and the sides of the pot removed. Soil from these pots was stored as the bulk soil samples.

### DNA Preparation and Sequencing

DNA was extracted using the MoBio Laboratories Power Soil DNA isolation kit according to the manufacturer’s instructions. Amplification of the V4 variable region of bacterial 16 s ribosomal DNA was performed essentially according to (Caporaso et al. 2012). Briefly, individual barcoded libraries are directly prepared by PCR using long primers incorporating the Illumina adapter sequences which allow large numbers of libraries to combined and sequenced on Illumina sequencers. In our case we prepared 123 libraries which we sequenced using a single 600 cycle Miseq version 3 kit, with the Miseq machine parameters set to produce a separate fastq file containing index reads.

### Sequence Analysis

Reads were processed and analyzed using scripts and programs from QIIME (Caporaso et al. 2010), Mothur (Schloss et al. 2009) and Phyloseq (McMurdie and Holmes 2013) among others. The paired reads were trimmed to 250 bp and joined using the join_paired_reads.py script in qiime. Reads were demultiplexed using the qiime_split_libraries_fastq.py script with a minimum phred quality score of 20. Reads were filtered to remove reads containing homopolymer stretches longer than 10 bases, ambiguous bases, reads less than 200 bp in length and reads longer than 275 bp using Mothur. OTU picking was performed using the open reference sumaclust/sortmerna method (Kopylova et al. 2012) as implemented in Qiime (pick_open_reference_otus.py). ChimeraSlayer (Haas et al. 2011) as implemented in Mothur was used with the greengenes database as reference (qiime_default_reference/gg_13_8_otus/rep_set_aligned/85_otus.fasta), and the representative phylogenetic tree was rebuilt based on the remaining OTUs after the chimeras were removed.

In order to compare the plant host phylogeny with the microbial phylogeny, chloroplast sequencing reads derived from whole genome sequences were mapped against the Nipponbare chloroplast genome using bwa (Li and Durbin 2009) and SNPs and Indels were called using bamtools (Barnett et al. 2011). The whole genome sequencing reads used are listed in Additional file 1: Table S1. A distance matrix was constructed using and a neighbor-joining tree constructed using the R package ape (Paradis et al. 2004). A similar neighbor-joining tree was constructed from a matrix of Bray-Curtis dissimilarities between rhizosphere samples.

To test for phylogenetic signal in the rhizosphere, scripts written by Easson and Thacker (Easson and Thacker 2014) were employed using a chloroplast phylogenetic tree constructed with the upgma method.

To test for differential representation of microbial taxa in different samples and sample groupings the Deseq2 package (Love et al. 2014) was used in conjunction with Phyloseq (McMurdie and Holmes 2013).

## Results and Discussion

### Wild *Oryza* Rhizosphere was Different to Cultivated Rice Rhizosphere in Complexity and in Composition

We surveyed 19 *Oryza* genotypes, six cultivated accessions and 11 wild species (Table 1). In lieu of a *O. longistamina* sample we used F1 hybrid seeds from a cross with *O. sativa* japonica Nipponbare, because of the difficulty of obtaining seed from self-incompatible *O. longistamina*. Sterilized dehulled seeds were sown in paddy field soil in pots in a greenhouse and grown for 4 weeks before sampling of loosely associated soil and combined material from the rhizosphere, rhizoplane and endosphere (hereafter referred to as the loosely attached soil and rhizosphere soil samples, respectively). Soil samples were also taken from unplanted pots at the same time and are referred to as bulk soil samples. Three replicate pots for each accession were planted with five seeds, and the pots were arranged in a randomized block design.

**Table 1:** Accessions and cultivars used in this study

Cultivar or Accession	Species	Genome type	Species complex
Nipponbare	*Oryza sativa*	AA	sativa
Taichung65	*Oryza sativa*	AA	sativa
93–11	*Oryza sativa*	AA	sativa
Davao	*Oryza sativa*	AA	sativa
Kasalath	*Oryza sativa*	AA	sativa
C0508	*Oryza glaberrima*	AA	sativa
W0120	*Oryza rufipogon*	AA	sativa
W1625	*Oryza meridionalis*	AA	sativa
W2199	*Oryza glumaepetula*	AA	sativa
W1588	*Oryza barthii*	AA	sativa
W1413F1	*Oryza longistaminata*	AA	sativa
W1514	*Oryza punctata*	BB	officinalis
W1213	*Oryza minuta*	BBCC	officinalis
W0002	*Oryza officinalis*	CC	officinalis
W1480B	*Oryza grandiglumis*	CCDD	officinalis
W0008	*Oryza australiensis*	EE	officinalis
W1711	*Oryza brachyantha*	FF	brachyantha
W1220	*Oryza longiglumis*	HHJJ	ridleyi
W0615	*Oryza granulata*	GG	meyeriana

### Differences Between Sample Types

**Fig. 1:**
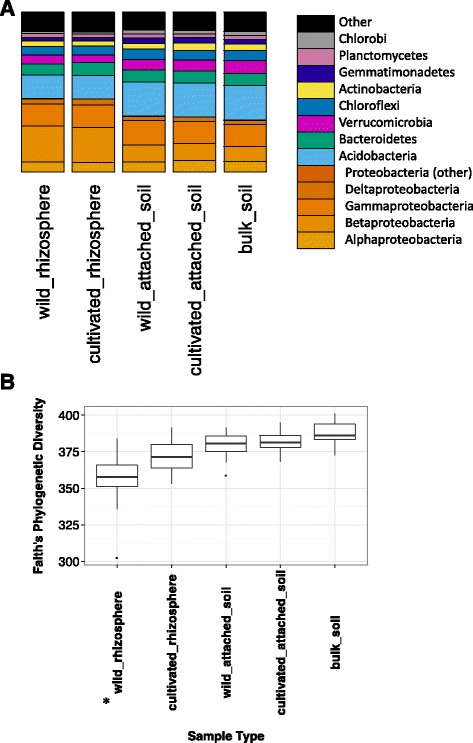
Summary of the represented taxa and their diversity in loosely attached and rhizosphere soil of cultivated and wild *Oryza* species. **a** — Bar graph showing the representation of the most highly represented phyla in the soils. Proteobacteria, as the most abundant phylum, are subdivided into classes. **b** — Boxplots representing the range of alpha diversity (Faith’s phylogenetic diversity) in loosely attached soil or rhizosphere of cultivated and wild *Oryza* species. Species diversity is reduced in the rhizosphere compared to the loose soil. Wild *Oryza* rhizosphere appears less diverse than the other sample types

In common with other studies of paddy field soil and *Oryza* rhizosphere, Proteobacteria, and Acidobacteria were dominant rhizosphere phyla, as well as Bacteriodetes, Chlorflexi and Verrucomicrobia (Knief et al. 2011, Edwards et al. 2015). In agreement with other studies, we found a reduction in alpha diversity in rhizosphere samples compared to loosely attached soil, due to selection and enrichment of a subset of bacterial species in the rhizosphere. On the broad scale, the rhizosphere had a higher proportion of Proteobacteria and reduced proportions of Acidobacteria and Chloroflexi compared with the loosely attached soil samples (Fig. 1a, Additional file 2: Table S2). The most obvious change was an increase in the proportion of Betaproteobacteria in the rhizosphere samples. Figure 1b shows a boxplot indicating the range of Faith’s phylogenic diversity (Faith et al. 2004) as computed using QIIME for soil or rhizosphere samples. Rhizosphere samples were reduced in diversity, possibly reflecting effects of the host plant roots on the microbial community in the rhizosphere. Loosely attached soil samples also showed a slight reduction in diversity compared to bulk soil, indicating that rhizosphere effects were not necessarily limited to our rhizosphere sample, but also extended into soil loosely adhered to the roots.

One of the motivations in this study was to compare wild and cultivated species or accessions to look for effects of domestication on root-associated microbial communities. Comparing alpha diversity (in this case Faith’s phylogenetic diversity) between wild and cultivated samples we observed that rhizosphere soils associated with wild *Oryza* species displayed lower alpha diversity than their cultivated rice counterparts (Fig. 1b). Furthermore, landraces (traditional or unimproved varieties) seemed to be intermediate between wild and cultivated samples Additional file 3: Figure S1A). Measures of alpha diversity for individual species and cultivars can be seen in Additional file 3: Figure S1B–D. Employing student’s *t*-test the rhizosphere associated with the wild *Oryza* species was significantly different to the other groups in terms of phylogenetic diversity (Additional file 4: Table S3), although the differences with landrace samples were not found to be significant.

**Fig. 2:**
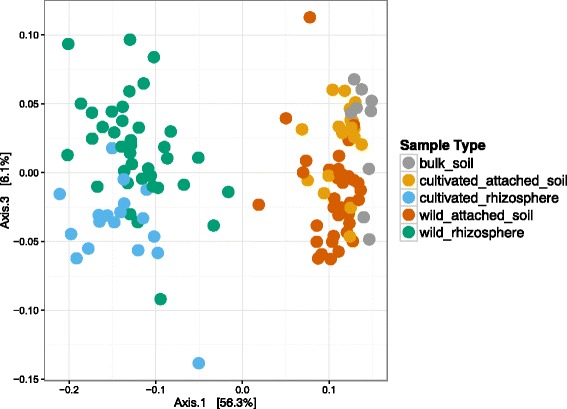
Ordination plots of the represented taxa in loosely attached and rhizosphere soil of cultivated and wild *Oryza* species showing differences in bacterial community composition between sample types. PCoA analysis of Unifrac distances calculated between all samples showing the differences in representation of taxa between the samples. Plot of the first and third PCoA axes colored to show the differences between the bacterial communities in rhizosphere and loosely attached soil fractions from wild and cultivated *Oryza* species

To consider differences in the composition of the bacterial communities associated with wild and cultivated rice roots, we used the QIIME pipeline to compute Unifrac distance metrics between the samples and to compare the samples using PCoA analysis. The different sample fractions were separated on the first PCoA axis, while the bacterial communities associated with cultivated and wild samples appeared to be separated on the third PCoA axis in rhizosphere samples, with a lesser but still noticeable difference in loosely attached soil samples (Fig. 2; a 3d representation of a plot of the PCoA analysis is available in Additional file 3: Figure S2).

We used PerMANOVA (implemented in the R-*vegan* function adonis) to distinguish the amount of variation explained by the sample fraction, the domestication status (wild or cultivated) or the interaction between these factors. Approximately 10 % of the total variation was explained by these factors, and sample fraction, domestication status and their interaction all made a significant contribution. Of these, the interaction between domestication status and sample fraction was the most important factor explaining 4.7 % of the variation (Additional file 5: Table S4). Thus it seems that there may be differences in the way that bacterial communities are distributed among sample fractions, depending on the domestication status of the host plants.

### Phylogeny of Root-Associated Microbiomes was Only Weakly Correlated with Oryza Phylogeny

Because all of the domesticated rice varieties possess the AA genome, and those species possessing other genomes are more distantly related, it is possible that the difference we observe between cultivated and wild species is not a result of domestication, and the distance separating their associated microbial communities simply reflects the phylogenetic distance of the host plants. A correlation of this kind, between the phylogenetic distances separating maize, wheat and sorghum and the Bray-Curtis dissimilarity separating their root-associated microbial communities, was found by Bouffaud et al. (2014).

On the other hand, if the rhizosphere composition has been under selection during the evolution of the *Oryza* genus, we might expect a lack of correlation between the genetic distance of the hosting plants and the magnitude of the distances between the microbial composition. We therefore attempted to estimate whether the difference in microbiota compositions showed a correlation with the phylogenetic relationships among the rice species and cultivars i.e. whether the difference in microbiota reflected the evolutionary divergence of the different rice species and cultivars. We first compared host (rice root) phylogeny with a distance-based (Bray-Curtis) phylogenetic tree constructed on the basis of differences between the root-associated microbial communities.

**Fig. 3:**
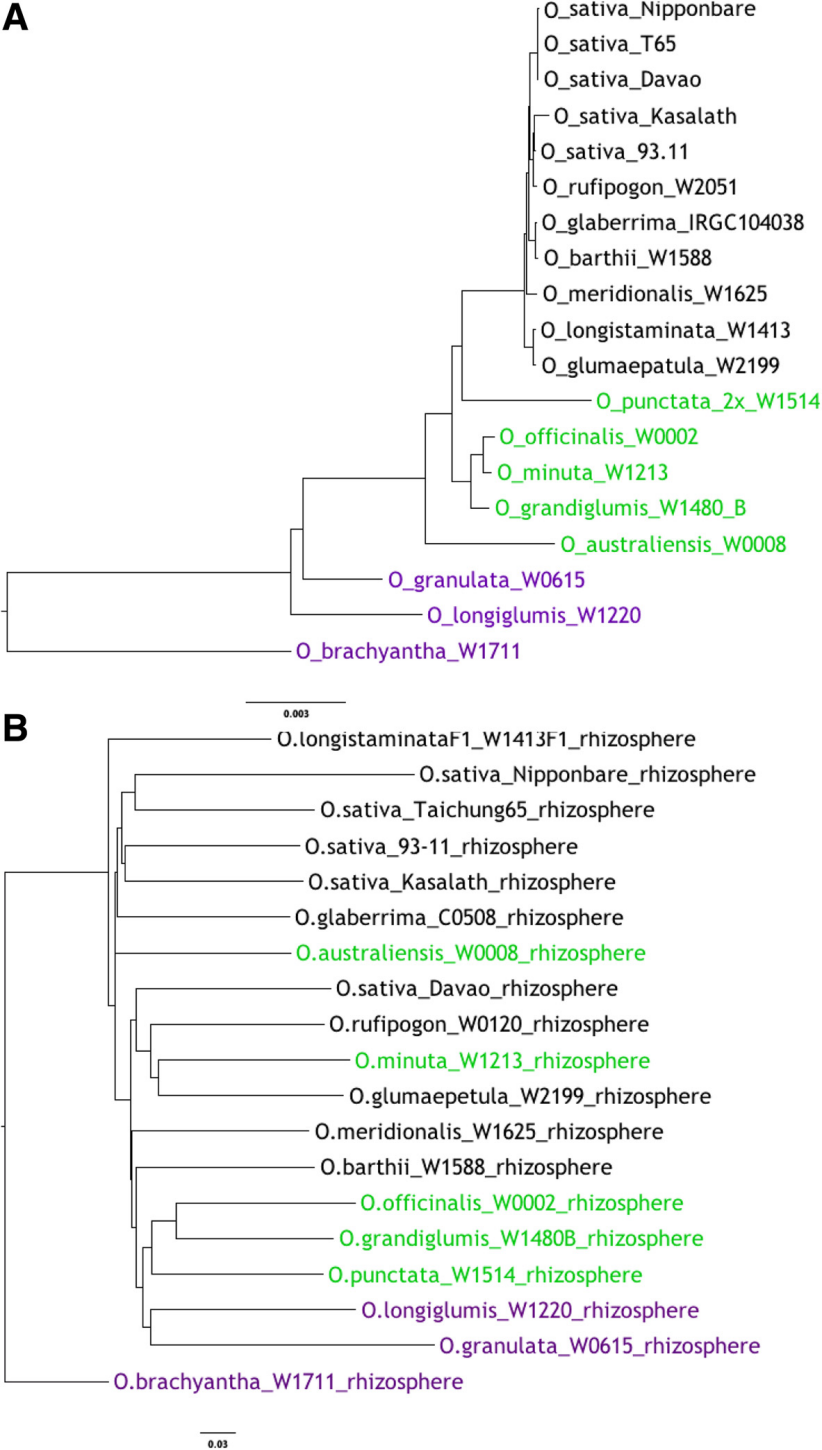
Phylogenetic relationships between *Oryza* species and cultivars and their associated microbiota. Comparison of phylogenies based on: **a** – Neighbor joining tree of *Oryza* species or cultivar chloroplast genome sequences; **b** – Neighbor joining tree based on Bray-Curtis dissimilarity between rhizosphere microbiota of the *Oryza* species and cultivars. Black text represents members of the *O. sativa* complex; green represents the *O. officinalis* complex. Early-diverged members of the genus appear in purple

To prepare a phylogenetic tree based on chloroplast sequences of the *Oryza* species we made use of chloroplast-derived reads from paired-end illumina whole-genome sequencing efforts. We mapped such reads onto the *O. sativa* Japonica Nipponbare chloroplast sequence, called SNPs and indels and constructed a neighbor-joining tree. We then constructed a similar neighbor-joining tree based on the Bray-Curtis dissimilarity between the rhizosphere communities associated with each cultivar or species and compared the topology to that of the *Oryza* genus. There was only limited congruence between the root-associated microbial communities of each species or accession and *Oryza* phylogeny (Fig. 3).

We also followed the methodology of Easson and Thacker (2014) to test for plant phylogenetic signal in the root-associated microbial communities. Phylogenetic signal means that more closely related *Oryza* species should share more similar root-associated microbial community compositions. We constructed a bacterial OTU phylogeny using the 16 s rDNA sequences from the 1500 most abundant bacterial OTUs in rhizosphere or soil samples using the default options of MAFFT and compared it with the chloroplast-based phylogeny of the Oryza species and accessions.

On this reduced dataset we calculated the Bray-Curtis dissimilarity (BCD, Beals 1984), the phylogenetic dissimilarity as measured by the mean nearest taxon distance (MNTD, Webb et al. 2008), and the Unifrac distance (Lozupone and Knight 2005) between the samples. We then used Mantel tests to assess the correlation between these distance measures and the plant host species identity and phylogeny.

We first used the adonis function in the vegan R package to test the effect of host species identity on BCD, MNTD and Unifrac distance. For the rhizosphere samples, adonis supported the hypothesis that the plant cultivar/species identity had a modest effect on the Bray-Curtis dissimilarity (df = 18, F = 1.92, *R*^2^ = 0.483, *P* < 0.001) and on the Unifrac distance (df = 18, F = 1.54, *R*^2^ = 0.428, *P* = 0.009), but the effect on the MNTD was not significant (df = 18, F = 1.88, *R*^2^ = 0.478, *P* = 0.087). Using Mantel tests, plant species/cultivar identity (*r* = 0.118, *P* < 0.001) and plant species/cultivar relatedness (*r* = 0.302, *P* = 0.017) each explained a small proportion of the variability in Bray-Curtis dissimilarity. Once cultivar identity was taken into account, the proportion of the variability in the BCD was reduced only slightly (*r* = 0.287, *P* = 0.015) suggesting that phylogenetic relatedness of the plant was more important than cultivar identity in affecting the Bray-Curtis dissimilarity of the rhizosphere microbiome, although in both cases the effect size is small. Considering the phylogenetic dissimilarity measures (MNTD and Unifrac), plant identity explained a very small proportion of the variability and plant relatedness did not explain a significant amount.

For the loosely attached soil fraction, a weak effect of plant cultivar identity was detected, but no significant correlation with the plant phylogeny for either BCD or phylogenetic dissimilarity (MNTD or Unifrac) was found. (See Additional file 6: Table S5).

### Differential Representation of Taxa in the Wild and Cultivated Rhizosphere

Given the apparent separation of cultivated and wild samples we further examined the differences in their root-associated microbiota. In order to examine what types of bacteria were differentially present, accounting for the difference between cultivated and wild varieties, we looked at differential representation between the cultivated and wild species. We conducted analysis of the differential abundance of OTUs in different samples by fitting a local regression model with a negative binomial distribution to the data and testing for differential abundance with a likelihood ratio test as implemented in the R package DESeq2 (Love et al. 2014) and in conjunction with the Phyloseq package (McMurdie and Holmes 2013).

Using a contrast between domesticated and wild rhizosphere, 423 OTUs were differentially represented between cultivated and wild rice rhizosphere out of a total number of 104517 taxa, 226 (0.2 %) higher in cultivated samples and 197 (0.19 %) higher in wild samples.

**Fig. 4:**
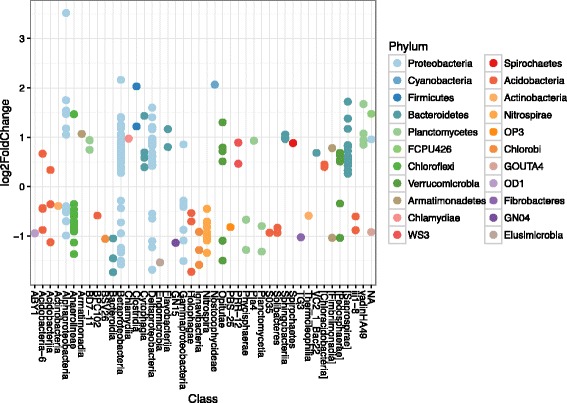
Differential representation of OTUs between wild *Oryza* species and domesticated cultivars and landraces. Differential abundance of OTUs in cultivated or wild rice rhizosphere was assessed by fitting a local regression model with a negative binomial distribution to the data and testing for differential abundance with a likelihood ratio test as implemented in the R package DESeq2 (Love et al. 2014) in conjunction with the Phyloseq package (McMurdie and Holmes 2013). Taxa are represented as dots in the graph of fold change. Positive values indicate higher representation in cultivated rice samples. Negative values indicate higher representation in wild rice samples. Samples with a p value less than 0.05 and a mean representation over all samples higher than 1 are shown

A graphical representation of the significantly differentially represented taxa (*P* < 0.05) with a base mean (mean of counts in all samples) greater than one is shown in Fig. 4 and a list of these significantly differentially represented taxa is presented in Additional file 7: Table S6. Of these, one hundred and twenty-eight OTUs were more highly represented in cultivated samples and 97 were higher in wild samples.

Notably several members of the Anaerolineae and Nitrospirae were decreased in cultivated rhizosphere relative to wild rhizosphere and none were decreased. On the other hand, several Saprospirae taxa were increased in cultivated rhizosphere and none decreased. Betaproteobacterial taxa were differentially present in both wild and cultivated samples. However, different families were either increased or decreased. Fifteen Comamondaceae taxa were reduced in the wild rice rhizosphere, and increased in the domesticated samples. Four Rhodocyclaceae taxa were reduced in cultivated samples and increased in wild samples (Additional file 7: Table S6).

In order to reduce any effect from phylogenetic distance of the host roots, and to compare a similar number of samples from wild and cultivated species/cultivars, we also made a similar comparison limited to the AA genome species (Additional file 3: Figure S3). We saw a similar pattern, with smaller numbers of significantly altered OTUs (Additional file 8: Table S7).

An intriguing possibility is a connection with the common symbiosis pathway, components of which are found in many plant species, and that is involved in connections with both mycorrhiza and nitrogen-fixing bacteria in legumes. Ikeda et al. (2011) showed that when this pathway is disrupted a number of changes occurred in the rhizosphere. Sphingomonadales, Rhizobiales, Rhodocyclales and Burkolderiales were reduced, while Anaerolinaeae and Clostridia were increased under field conditions. In our differential representation analysis taxa belonging to the Anaerolineae increased in wild vs cultivated rhizosphere. Furthermore, Sphingomonadales taxa are found among significantly downregulated Alphaproteobacteria and Burkholderiales are found among downregulated Betaproteobacteria, whether comparing all samples, or only those with the AA genome. It is tempting to speculate that in selecting for improved yield and growth during domestication and breeding, factors affecting symbiotic pathways may have been selected.

### Differentiated Rhizosphere Microbiome in the Earliest Diverged Wild Species

**Fig. 5:**
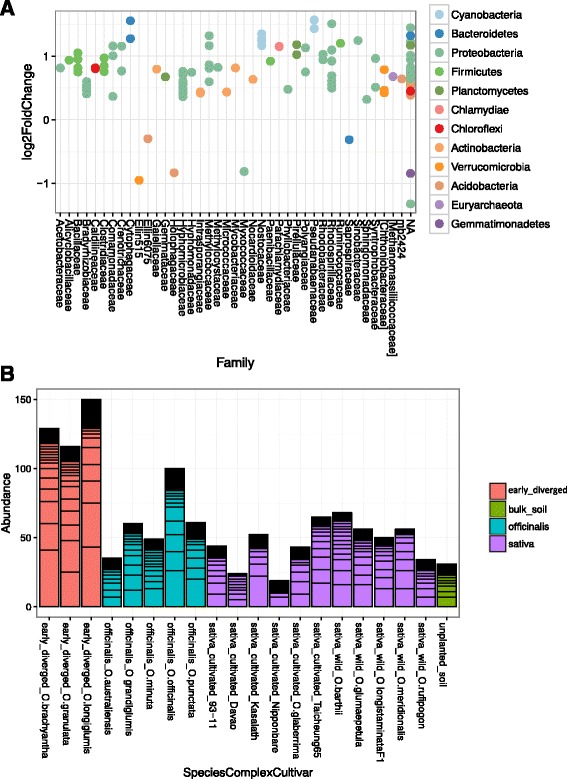
Differential representation in the rhizosphere of early-diverged *Oryza* species. **a** — Differential abundance of OTUs in the earliest diverged *Oryza* species rhizosphere was assessed by fitting a local regression model with a negative binomial distribution to the sequence count data and testing for differential abundance with a likelihood ratio test as implemented in the R package DESeq2 (Love et al. 2014) in conjunction with the Phyloseq package (McMurdie and Holmes 2013). Taxa are represented as dots in the graph of fold change. Positive values indicate higher representation in early-diverged species samples. Samples with a *p* value less than 0.05 and a mean representation over all samples higher than 1. **b** — Several taxa that are Type I (obligate) and Type II (facultative) methanotrophs of the families Methylococcaceae and Methylocystaceae respectively were more common in the rhizosphere of the earliest diverged species of the genus (*O. brachyantha, O. granulata* and *O. longiglumis*). The bar graph represents the total reads for the genus Methylomonas for each species/cultivar from a randomly selected 10000 reads from each sample

We looked for differential representation of taxa associated with individual cultivars or species. There was significant overlap between replicates of individual cultivars in PcoA of Unifrac distances, so that it was difficult to identify specific features for individual accessions. Additional file 3: Figure S4A shows a PCoA plot of the rhizosphere samples labeled by individual cultivar. However, we noted that those species in the “early-diverged” part of the genus possessing FF, GG and HHJJ genomes appeared separate on the third PcoA axis (Additional file 3: Figure S4B). Analyzing the differential abundance of OTUs for this group showed that they were enriched in several taxa belonging to the Alphaproteobacteria, including Sphingomonaceae, Bradyrhizobiaceae, Methylocystaceae, Rhodospirillaceae and Hyphomicrobiaceae; and Methylococcales of the Gammaproteobacteria (Fig. 5a; Additional file 9: Table S8). Some OTUs representing methylotrophs of both type I (obligate), and type II (facultative) seem to be enriched in the rhizosphere of *O. brachyantha*, *O. longiglumis* and *O. ridleyi* (Fig. 5a), compared to the other species. In particular, Methylomonas are highly represented and differentially present in these three early-diverged species of the *Oryza* genus (Fig. 5b).

## Conclusion

Wild rice rhizosphere bacterial communities differed in complexity (species richness) and composition compared with cultivated rice rhizospheres. Wild rhizosphere was enriched in selected taxa belonging to the Anaerolineae and Nitrospirae and depleted in Saprospirae taxa, compared with cultivated rhizosphere, although the size of the effect was small in the context of the whole rhizosphere. Parallels with the effect of removing an element of the common symbiosis pathway may suggest that factors acting on that pathway may have been selected for during domestication and breeding. Such elements of rhizosphere composition might be responsible for improved yield or disease resistance.

Distances between rhizosphere communities did not correlate well with the genetic distances of the plant hosts. This is in contrast to the results found by (Bouffaud et al. 2014) and may suggest an influence of selection on rhizosphere communities.

Species in the early-diverged part of the genus, *O. brachyantha*, *O. longiglumis* and *O. granulata* have increased counts of certain methanotrophs in their rhizosphere. This is of interest because methane emission from rice paddies makes a significant contribution to the greenhouse effect. If rice plants could be bred to optimize the community of methanotrophs in and around rice roots it could be possible to reduce the damage caused by emissions resulting from food production. Introgression of genetic material from these wild species into cultivated rice varieties may present opportunities for the manipulation of rhizosphere functions.

## Additional files

Additional file 1: Table S1. Sequencing data used for chloroplast sequence based phylogenies. (XLSX 39 kb) Additional file 2: Table S2. Kruskal-Wallis test of differently represented bacterial classes in different sample types. (XLSX 87 kb) Additional file 3: Figures S1-S4. Figure S1. Measures of alpha diversity. Figure S2. PCoA plot of Unifrac distances coloured by sample type. Figure S3. Differential representation of taxa in cultivated or wild rhizosphere in AA genome species. Figure S4. PCoA plot of rhizosphere sample Unifrac distances colored by cultivar/accession (A) or species complex/genus section (B). (PPTX 544 kb) Additional file 4: Table S3. Comparison of alpha diversity (Faith’s phylogenetic diversity) among sample types. (PPTX 92 kb) Additional file 5: Table S4. PerMANOVA analysis to determine the proportion of variation among bacterial samples explained by sample fraction, domestication status, and the interaction between them. (PPTX 121 kb) Additional file 6: Table S5. Detection of phylogenetic signal in root-associated microbial communities —  Mantel tests to assess the correlation between bacterial community distance measures and the plant host species identity. (XLSX 66 kb) Additional file 7: Table S6. Differentially represented taxa in cultivated vs wild rhizosphere. (XLSX 78 kb) Additional file 8: Table S7. Differential representation of Taxa in AA genome cultivated vs wild rhizosphere. (XLSX 66.0 kb) Additional file 9: Table S8. Differential representation (over or underrepresentation) in rhizosphere of early-diverged Oryza species. (XLSX 10 kb) Additional file 10. OTU table for 16S rDNA sequencing data in biom format. (BIOM 44979 kb) Additional file 11. Sample metadata table corresponding to OTU table in Additional file 10. (TXT 43 kb) Additional file 12. Chloroplast sequence variant data for Oryza species and cultivars in vcf format. (VCF 17719 kb)

## References

[CR1] Barnett DW, Garrison EK, Quinlan AR (2011). BamTools: a C++ API and toolkit for analyzing and managing BAM files. Bioinformatics.

[CR2] Beals EW (1984) Bray-Curtis ordination: an effective strategy for analysis of multivariate ecological data. Adv Ecol Res 14:1–55. doi:10.1016/S0065-2504(08)60168-3

[CR3] Berg G, Smalla K (2009). Plant species and soil type cooperatively shape the structure and function of microbial communities in the rhizosphere. FEMS Microbiol Ecol.

[CR4] Bouffaud M-L, Poirier M-A, Muller D, Moënne-Loccoz Y (2014). Root microbiome relates to plant host evolution in maize and other Poaceae. Environ Microbiol.

[CR5] Bulgarelli D, Rott M, Schlaeppi K (2012). Revealing structure and assembly cues for Arabidopsis root-inhabiting bacterial microbiota. Nature.

[CR6] Bulgarelli D, Garrido-Oter R, Münch PC (2015). Structure and Function of the Bacterial Root Microbiota in Wild and Domesticated Barley. Cell Host Microbe.

[CR7] Caporaso JG, Kuczynski J, Stombaugh J (2010). QIIME allows analysis of high-throughput community sequencing data. Nat Methods.

[CR8] Caporaso JG, Lauber CL, Walters WA (2012). Ultra-high-throughput microbial community analysis on the Illumina HiSeq and MiSeq platforms. ISME J.

[CR9] Carvalhais LC, Dennis PG, Badri DV (2015). Linking Jasmonic Acid Signaling, Root Exudates, and Rhizosphere Microbiomes. Mol Plant Microbe Interact.

[CR10] Easson CG, Thacker RW (2014). Phylogenetic signal in the community structure of host-specific microbiomes of tropical marine sponges. Front Microbiol.

[CR11] Edwards J, Johnson C, Santos-Medellín C, et al (2015) Structure, variation, and assembly of the root-associated microbiomes of rice. Proc Natl Acad Sci 201414592. doi: 10.1073/pnas.1414592112 10.1073/pnas.1414592112 PMC4345613 25605935

[CR12] Faith DP, Reid CM, Hunter J (2004). Integrating Phylogenetic Diversity, Complementarity, and Endemism for Conservation Assessment. Conserv Biol.

[CR13] Haas BJ, Gevers D, Earl AM (2011). Chimeric 16S rRNA sequence formation and detection in Sanger and 454-pyrosequenced PCR amplicons. Genome Res.

[CR14] Hartmann A, Rothballer M, Schmid M (2008). Lorenz Hiltner, a pioneer in rhizosphere microbial ecology and soil bacteriology research. Plant Soil.

[CR15] Hiltner L (1904). Über neuere Erfahrungen und Probleme auf dem Gebiet der Bodenbackteriologie und unter besonderer Berücksichtigung der Gründüngung und Brache. Arb Dtsch Landwirtsch Ges.

[CR16] Ikeda S, Okubo T, Takeda N, et al (2011) OsCCaMK genotype determines bacterial communities in rice roots under paddy and upland field conditions. Appl Environ Microbiol AEM.00315-11. doi: 10.1128/AEM.00315-11 10.1128/AEM.00315-11 PMC3127688 21551283

[CR17] Jacquemin J, Ammiraju JSS, Haberer G (2013). 15 MYA of evolution in the Oryza genus shows extensive gene family expansion. Mol Plant.

[CR18] Knief C, Delmotte N, Chaffron S (2011). Metaproteogenomic analysis of microbial communities in the phyllosphere and rhizosphere of rice. ISME J.

[CR19] Kopylova E, Noé L, Touzet H (2012). SortMeRNA: fast and accurate filtering of ribosomal RNAs in metatranscriptomic data. Bioinformatics.

[CR20] Kurata N (2008). Rice Biology in the Genomics Era III.2 Chromosome and Genome Evolution in Rice.

[CR21] Lakshmanan V, Selvaraj G, Bais HP (2014). Functional Soil Microbiome: Belowground Solutions to an Aboveground Problem. Plant Physiol.

[CR22] Li H, Durbin R (2009). Fast and accurate short read alignment with Burrows–Wheeler transform. Bioinformatics.

[CR23] Love MI, Huber W, Anders S (2014). Moderated estimation of fold change and dispersion for RNA-seq data with DESeq2. Genome Biol.

[CR24] Lozupone C, Knight R (2005). UniFrac: a New Phylogenetic Method for Comparing Microbial Communities. Appl Environ Microbiol.

[CR25] Lundberg DS, Lebeis SL, Paredes SH (2012). Defining the core Arabidopsis thaliana root microbiome. Nature.

[CR26] McMurdie PJ, Holmes S (2013). phyloseq: An R Package for Reproducible Interactive Analysis and Graphics of Microbiome Census Data. PLoS ONE.

[CR27] Paradis E, Claude J, Strimmer K (2004). APE: Analyses of Phylogenetics and Evolution in R language. Bioinformatics.

[CR28] Peiffer JA, Spor A, Koren O (2013). Diversity and heritability of the maize rhizosphere microbiome under field conditions. Proc Natl Acad Sci.

[CR29] Quiza L, St-Arnaud M, Yergeau E (2015). Harnessing phytomicrobiome signaling for rhizosphere microbiome engineering. Front Plant Sci.

[CR30] Schloss PD, Westcott SL, Ryabin T (2009). Introducing mothur: Open-Source, Platform-Independent, Community-Supported Software for Describing and Comparing Microbial Communities. Appl Environ Microbiol.

[CR31] Szoboszlay M, Lambers J, Chappell J (2015). Comparison of root system architecture and rhizosphere microbial communities of Balsas teosinte and domesticated corn cultivars. Soil Biol Biochem.

[CR32] Vaughan DA, Ge S, Kaga A, Tomooka N (2008) Rice Biology in the Genomics Era III.1 Phylogeny and biogeography of the genus Oryza. Springer, Heidelberg, pp 219–234

[CR33] Webb CO, Ackerly DD, Kembel SW (2008). Phylocom: software for the analysis of phylogenetic community structure and trait evolution. Bioinformatics.

