# Supplementary material for: Effect of Wild and Cultivated Rice Genotypes on Rhizosphere Bacterial Community Composition
Source: Rice (N Y). 2016 Aug 24;9(1):42. doi: 10.1186/s12284-016-0111-8 (PMC4996804; doi:10.1186/s12284-016-0111-8)
Supplement: Additional file 3: Figures S1-S4. — Figure S1. Measures of alpha diversity. Figure S2. PCoA plot of Unifrac distances coloured by sample type. Figure S3. Differential representation of taxa in cultivated or wild rhizosphere in AA genome species. Figure S4. PCoA plot of rhizosphere sample Unifrac distances colored by cultivar/accession (A) or species complex/genus section (B). (PPTX 544 kb) [file 12284_2016_111_MOESM3_ESM.pptx]

## Slide 1
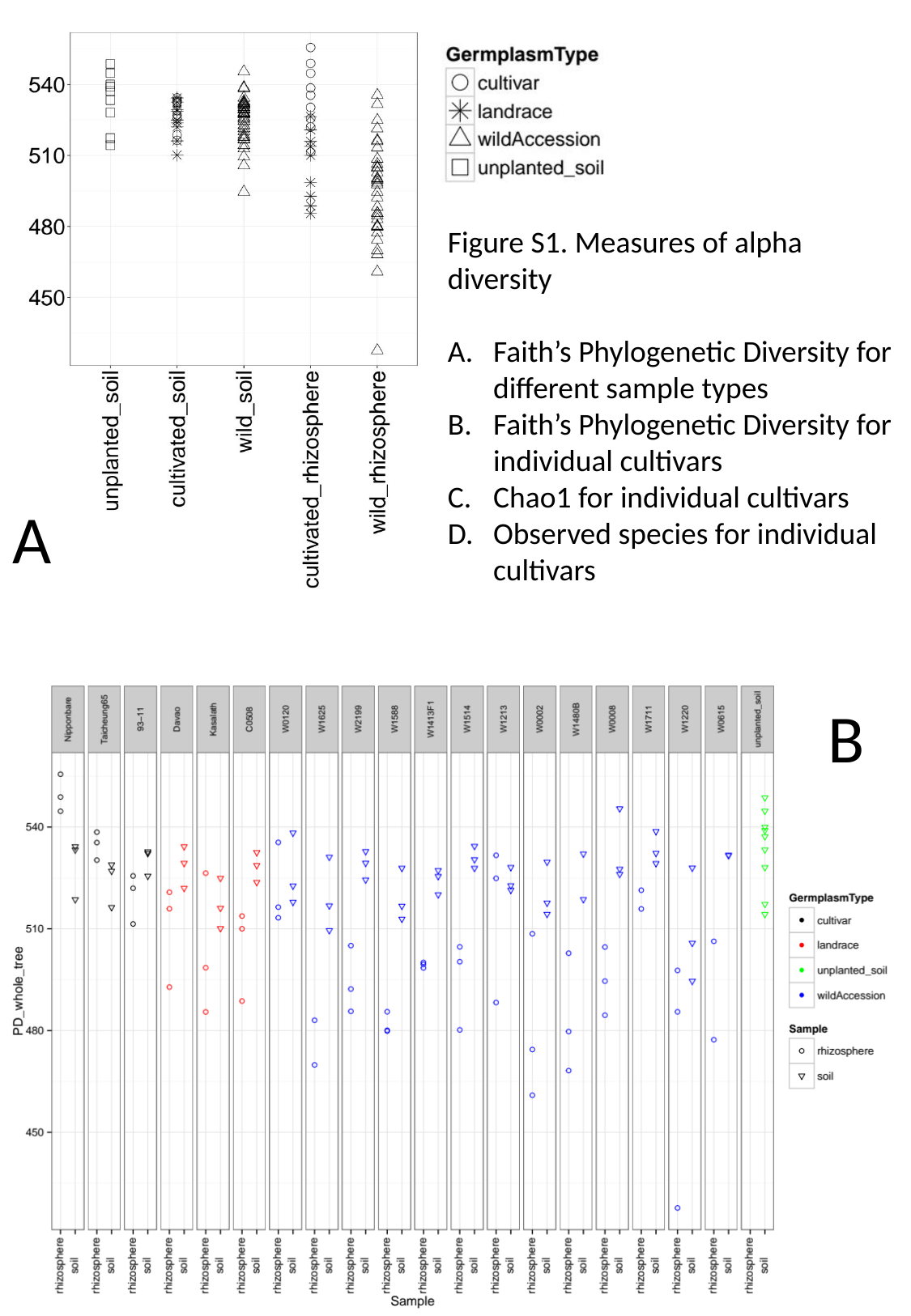

Figure S1. Measures of alpha diversity
Faith’s Phylogenetic Diversity for different sample types
Faith’s Phylogenetic Diversity for individual cultivars
Chao1 for individual cultivars
Observed species for individual cultivars
A
B

## Slide 2
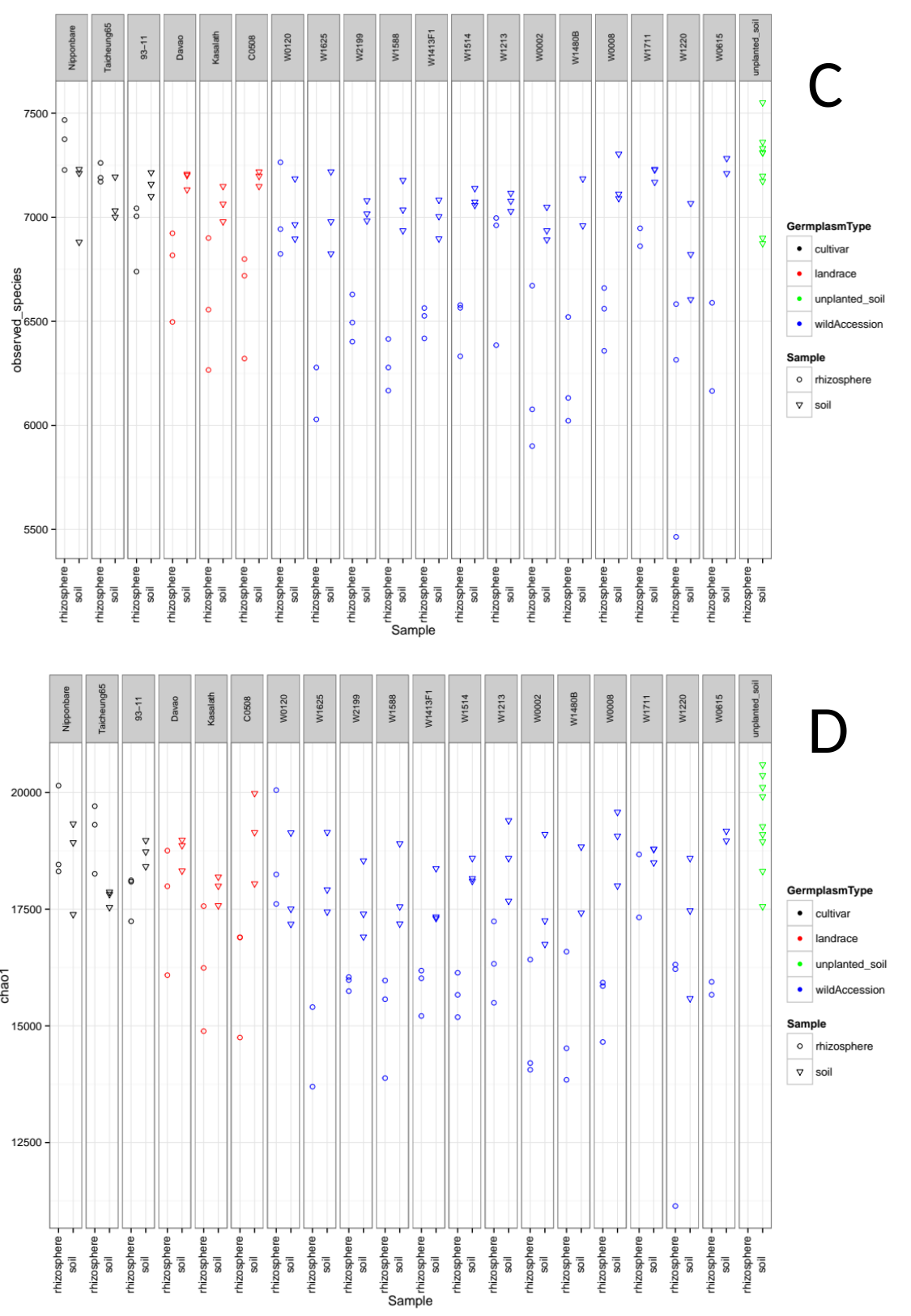

C
D

## Slide 3
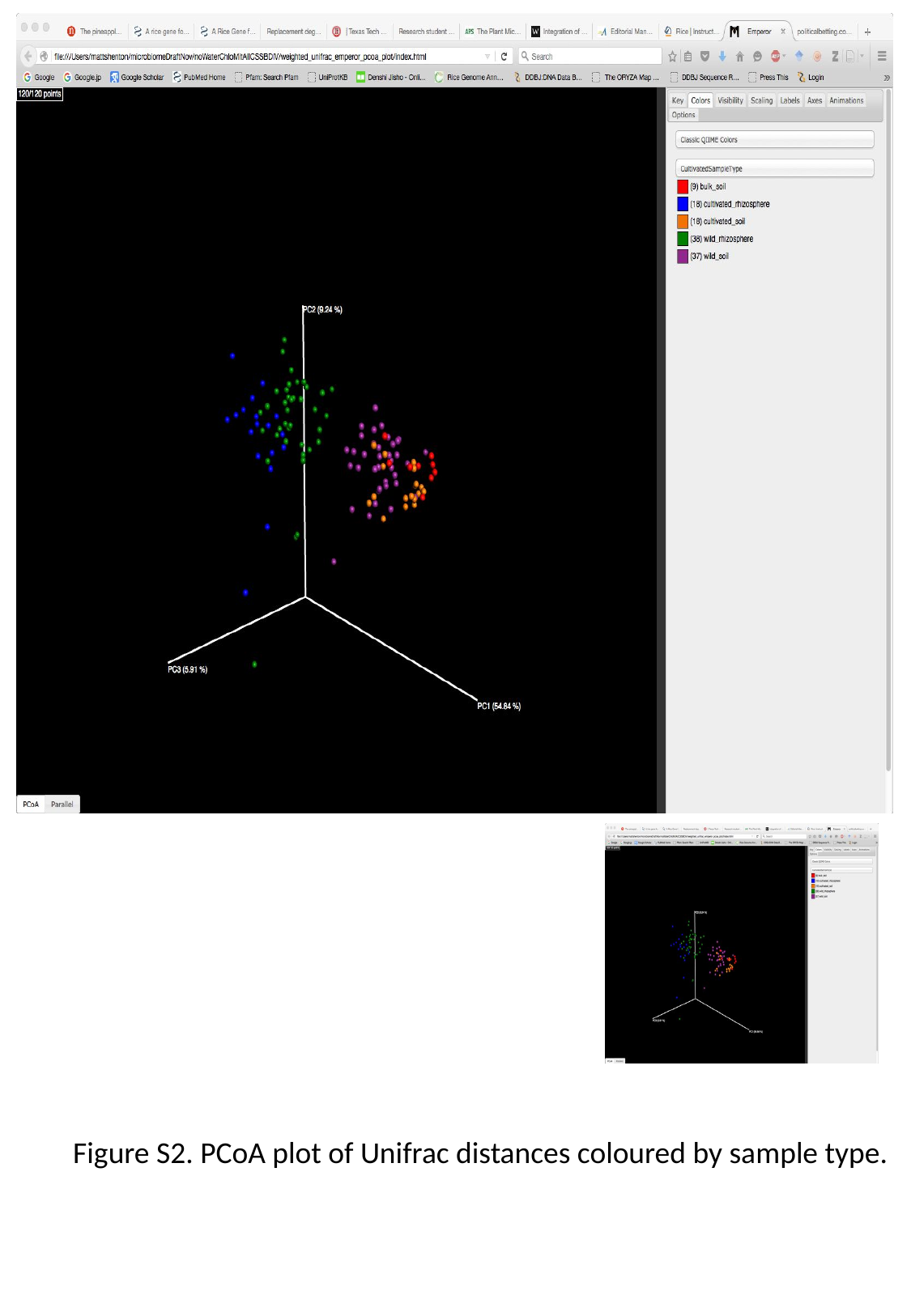

Figure S2. PCoA plot of Unifrac distances coloured by sample type.

## Slide 4
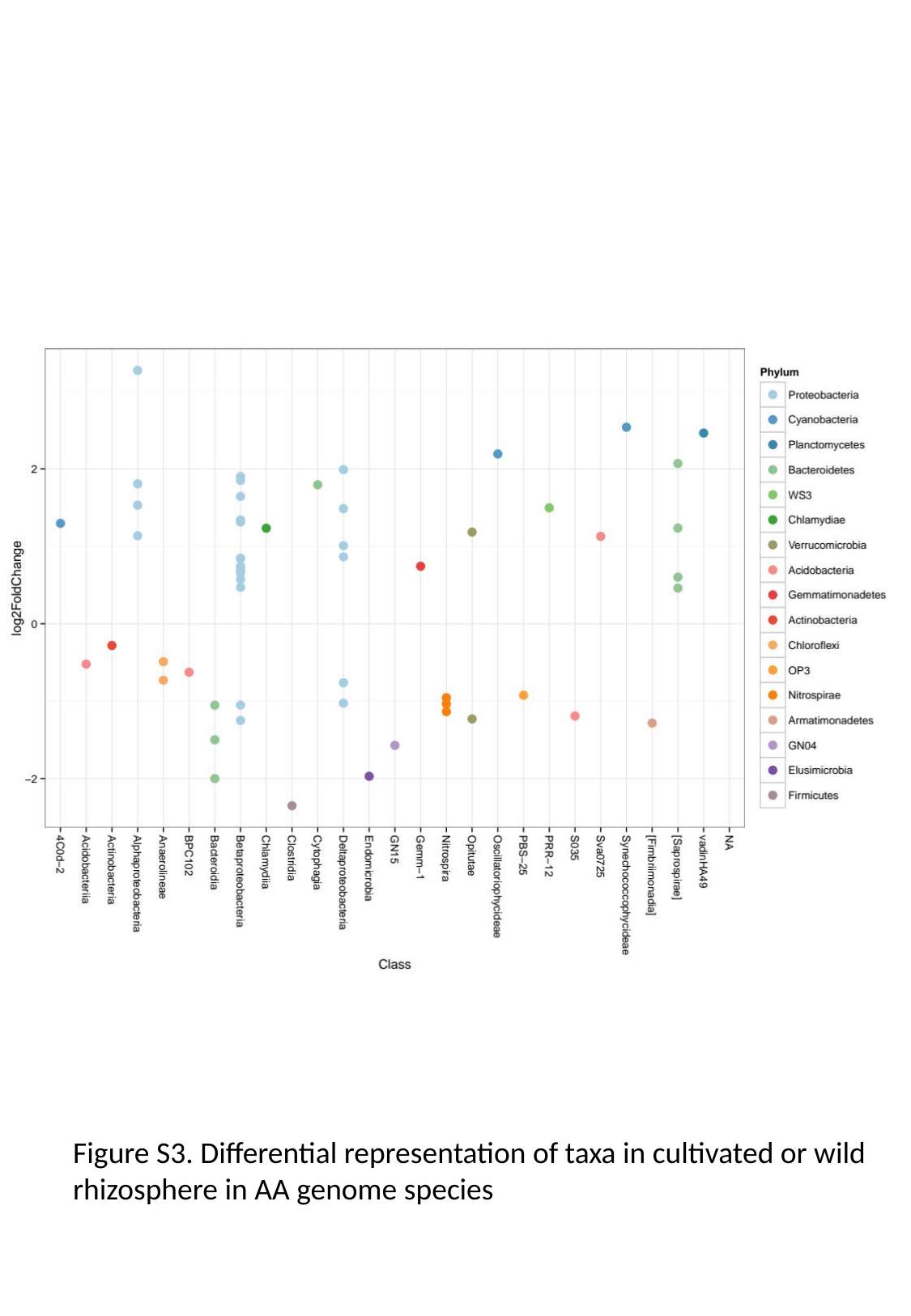

Figure S3. Differential representation of taxa in cultivated or wild rhizosphere in AA genome species

## Slide 5
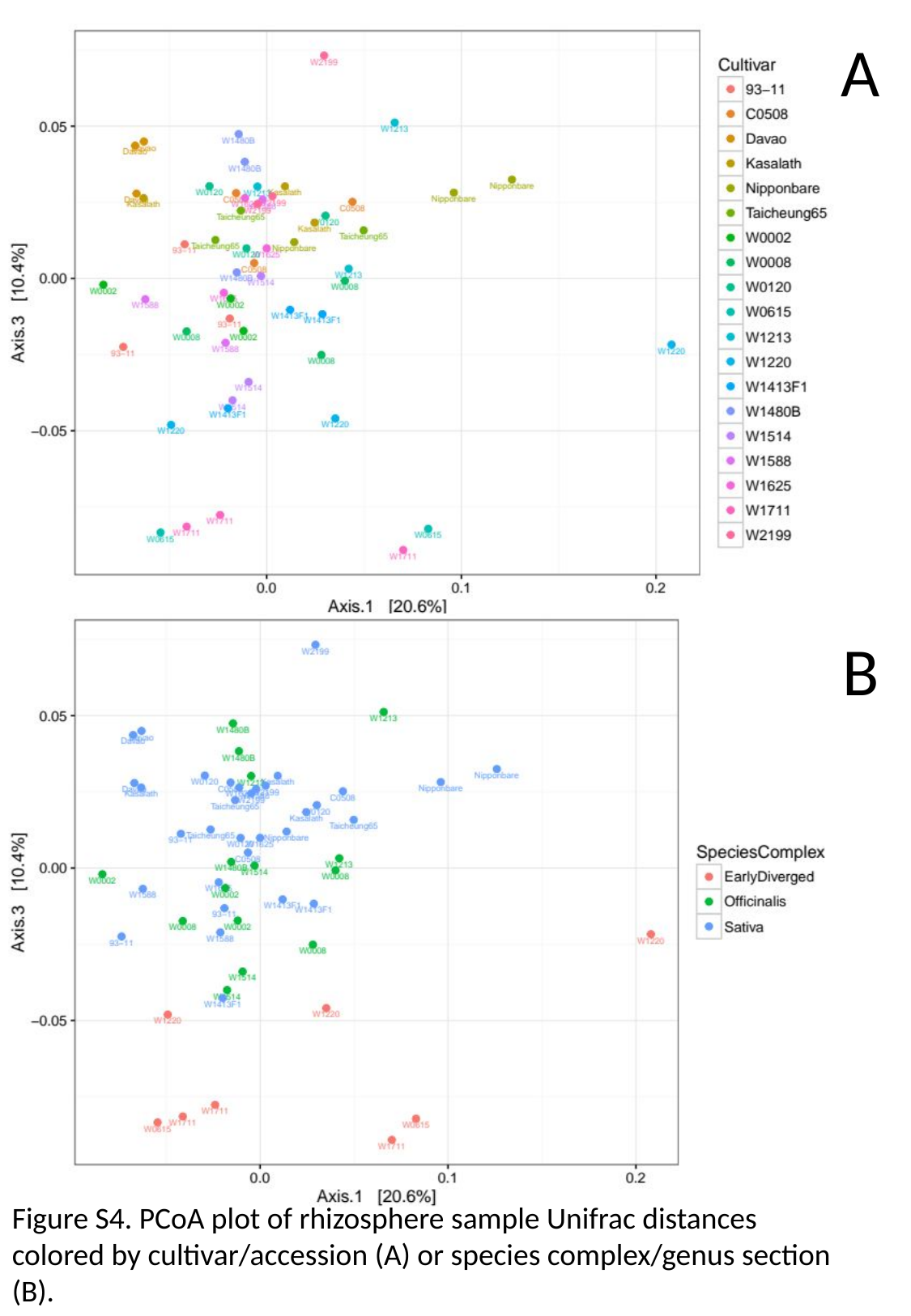

A
B
Figure S4. PCoA plot of rhizosphere sample Unifrac distances colored by cultivar/accession (A) or species complex/genus section (B).
