# Supplementary material for: Effect of Wild and Cultivated Rice Genotypes on Rhizosphere Bacterial Community Composition
Source: Rice (N Y). 2016 Aug 24;9(1):42. doi: 10.1186/s12284-016-0111-8 (PMC4996804; doi:10.1186/s12284-016-0111-8)
Supplement: Additional file 4: Table S3. — Comparison of alpha diversity (Faith’s phylogenetic diversity) among sample types. (PPTX 92 kb) [file 12284_2016_111_MOESM4_ESM.pptx]

## Slide 1
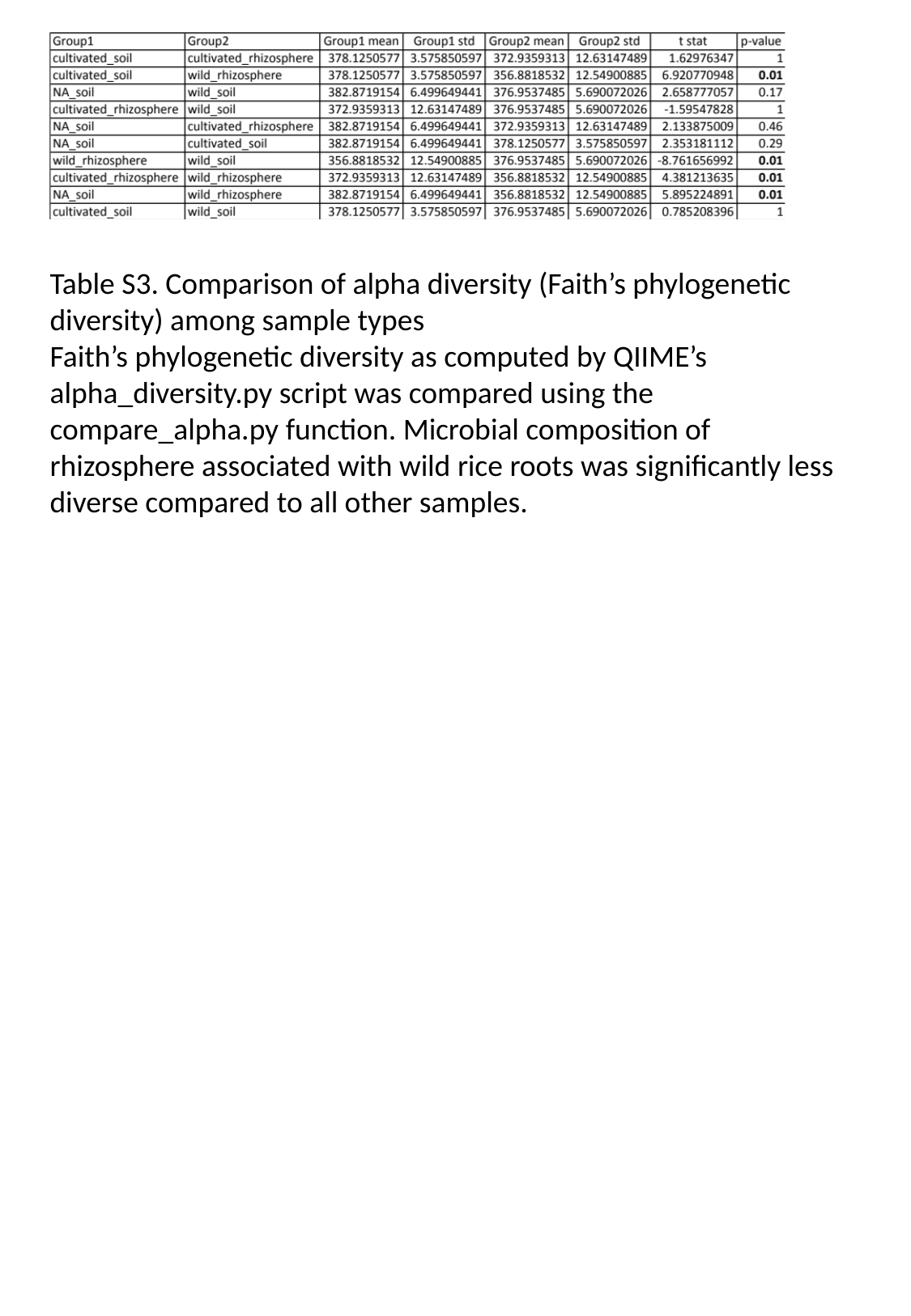

Table S3. Comparison of alpha diversity (Faith’s phylogenetic diversity) among sample types
Faith’s phylogenetic diversity as computed by QIIME’s alpha_diversity.py script was compared using the compare_alpha.py function. Microbial composition of rhizosphere associated with wild rice roots was significantly less diverse compared to all other samples.
