# Supplementary material for: Effect of Wild and Cultivated Rice Genotypes on Rhizosphere Bacterial Community Composition
Source: Rice (N Y). 2016 Aug 24;9(1):42. doi: 10.1186/s12284-016-0111-8 (PMC4996804; doi:10.1186/s12284-016-0111-8)
Supplement: Additional file 5: Table S4. — PerMANOVA analysis to determine the proportion of variation among bacterial samples explained by sample fraction, domestication status, and the interaction between them. (PPTX 121 kb) [file 12284_2016_111_MOESM5_ESM.pptx]

## Slide 1
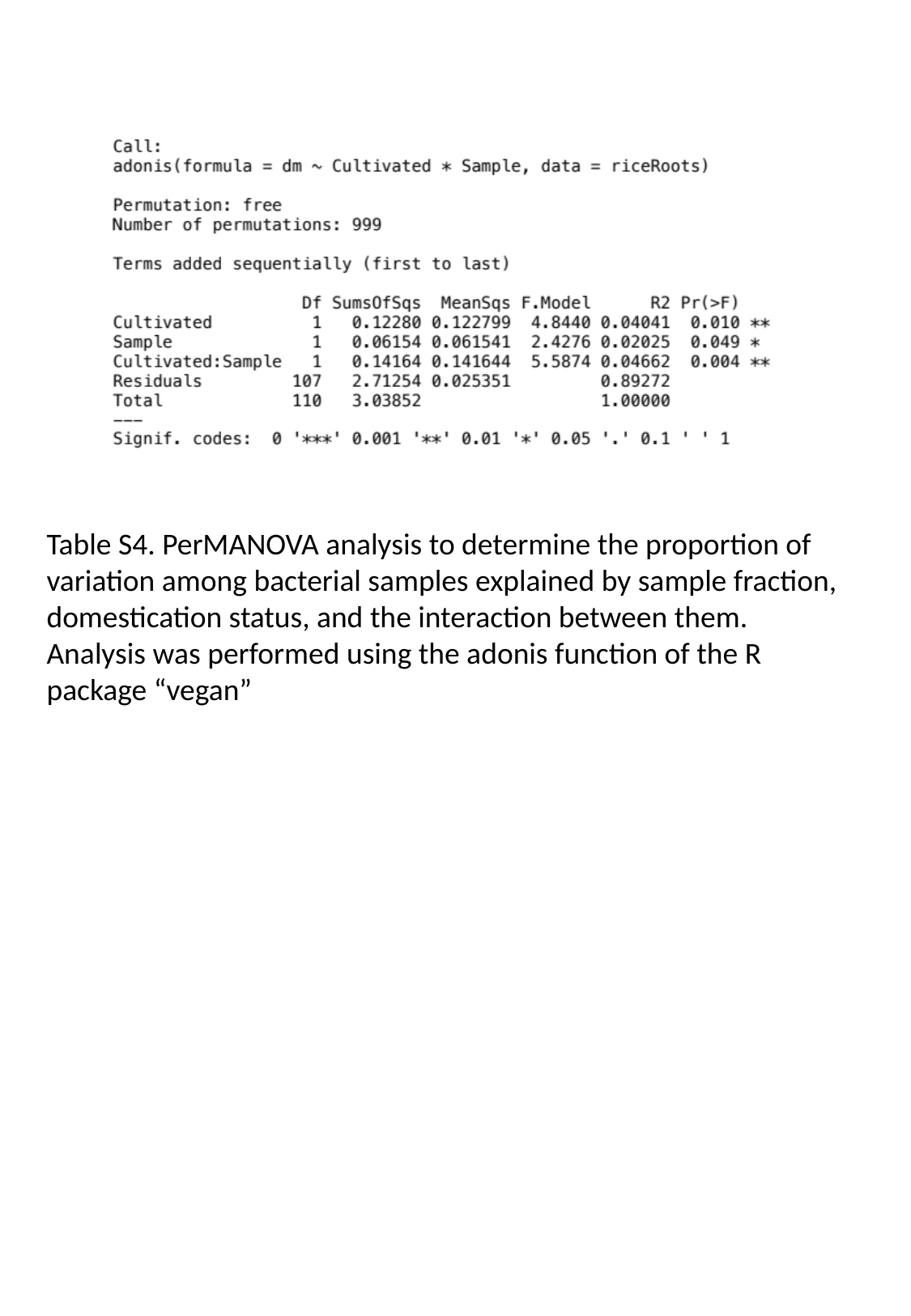

Table S4. PerMANOVA analysis to determine the proportion of variation among bacterial samples explained by sample fraction, domestication status, and the interaction between them. Analysis was performed using the adonis function of the R package “vegan”
